# Small cell carcinoma in ulcerative colitis - new treatment option: a case report

**DOI:** 10.1186/1477-7819-8-100

**Published:** 2010-11-18

**Authors:** Christoforos Kosmidis, Christoforos Efthimiadis, Georgios Anthimidis, Kalliopi Vasiliadou, Ioanna Tzeveleki, Panagiotis Fotiadis, Georgios Basdanis

**Affiliations:** 1 Department of Surgery, Interbalkan European Medical Center, Thessaloniki, Greece

## Abstract

**Background:**

The most common type of carcinoma associated with ulcerative colitis (UC) is adenocarcinoma. We present a case of primary rectal small cell carcinoma in a patient with a history of UC.

**Methods:**

A 34-year-old male diagnosed with UC for 10 years was not consistent with the usual annual follow-up and presented with mucoid-bloody diarrhea. Colonoscopy revealed a rectal mass 2 cm distant from the anal verge. The patient underwent a total proctocolectomy with preservation of the anal sphincters, construction of an ileal reservoir, anastomosis of the reservoir to the anus (J configuration) and protective loop ileostomy.

**Results:**

Histological examination showed undifferentiated small cell carcinoma.

**Conclusions:**

This is the first case of small cell carcinoma in a background of UC reported to be treated surgically and the patient and has no reccurence 18 months postoperatively.

## Background

Primary small cell carcinoma (SCC) of the colon and the rectum is very rare, with an incidence of less than 0,2% of all colorectal cancers[1]. The most common histological type of carcinoma associated with ulcerative colitis is adenocarcinoma[2]. We present a case of primary rectal small cell carcinoma in a patient with a history of ulcerative colitis, which is the fifth case reported and the first treated surgically.

## Methods

### Case presentation

**Figure 1:**
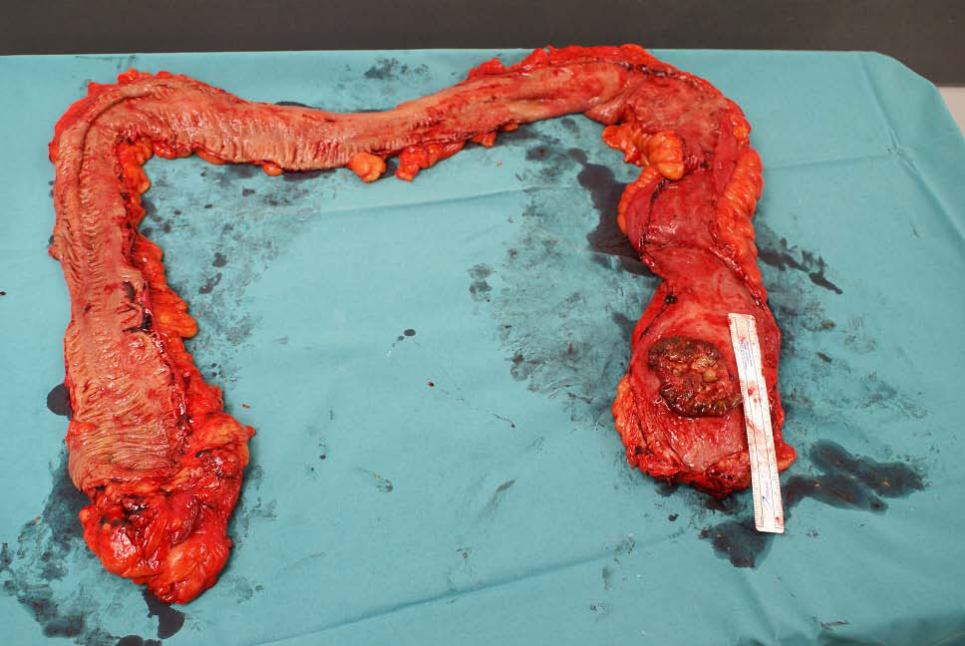
**Total proctocolectomy specimen with ulcerative colitis and small cell carcinoma in the inferior part of the rectum**.

**Figure 2:**
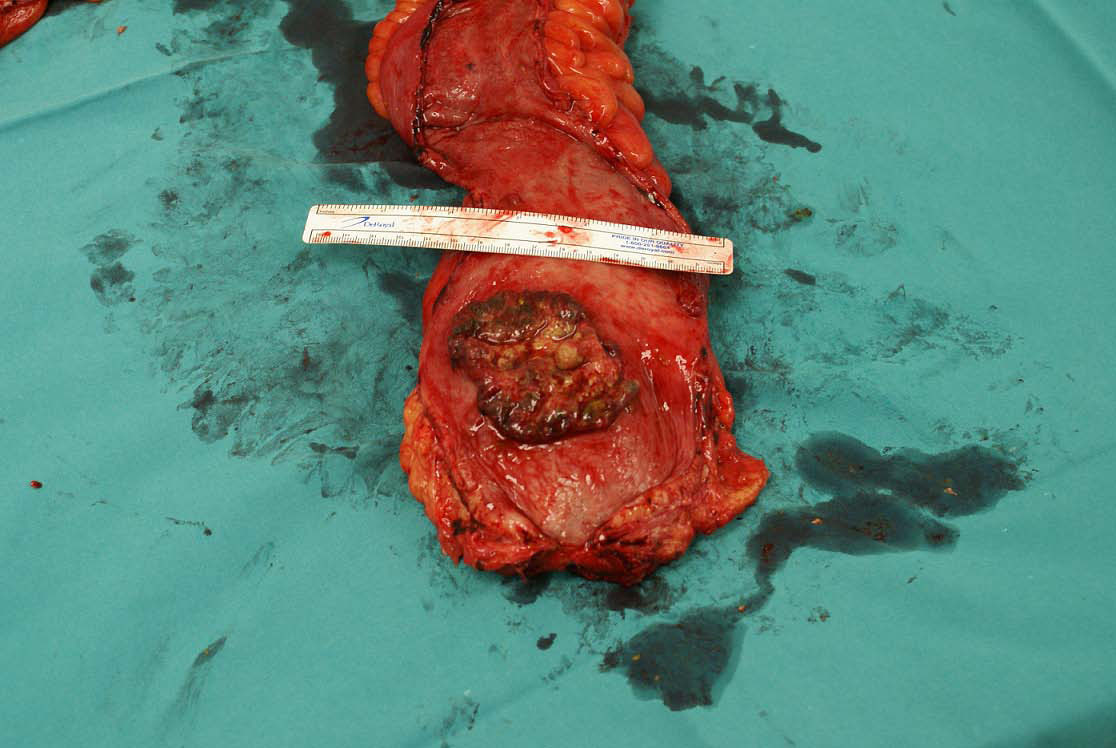
**Magnification of the small cell carcinoma in the inferior part of the rectum**.

A 34-year-old male diagnosed with ulcerative colitis for 10 years presented with mucoid-bloody diarrhea and none extraintestinal manifestation. His haemoglobin was 10,6 gr/dl. The patient had been prescribed methylprednisolone 24 gr daily during the last years, but he was not consistent with the usual annual follow-up. A colonoscopy was immediately performed and revealed a rectal mass 2 cm distant from the anal verge.. Biopsy results of the colonoscopy showed an undifferentiated small cell carcinoma positive to Thyroid transcription factor-1 (TTF-1). Subsequently a primary location in the lung was also examined Magnetic resonance imaging (MRI) scan confirmed that finding by demonstrating a rectal tumor extending between 2 cm proximally to the anal verge and 7 cm in the rectal canal, and enlarged adjacent lymph nodes. Abdominal, chest and brain computerized tomography (CT) showed no metastatis. The patient underwent a total proctocolectomy with preservation of the anal sphincters, construction of an ileal reservoir, anastomosis of the reservoir to the anus (J configuration) and protective loop ileostomy (Figures 1 and 2).

## Results

**Figure 3:**
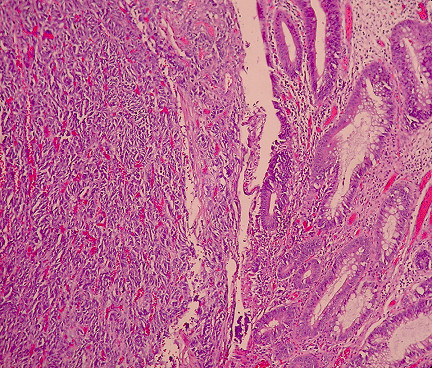
**Small cell carcinoma with invasion of the mucosa (H+E ×100)**.

**Figure 4:**
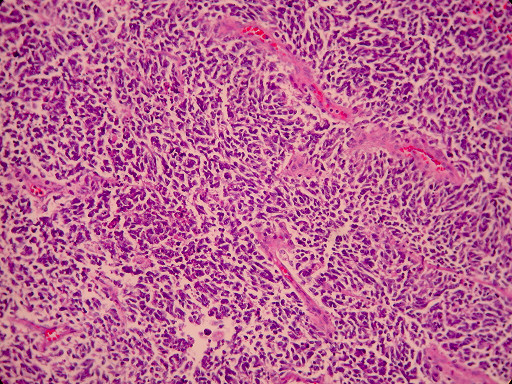
**Small cell carcinoma in deeper invasion (H+E ×200)**.

**Figure 5:**
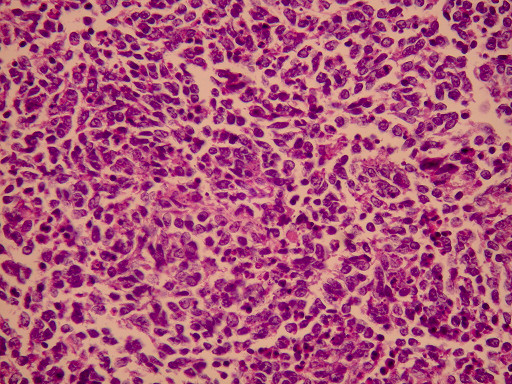
**Small cell carcinoma in deeper invasion (H+E ×400)**.

**Figure 6:**
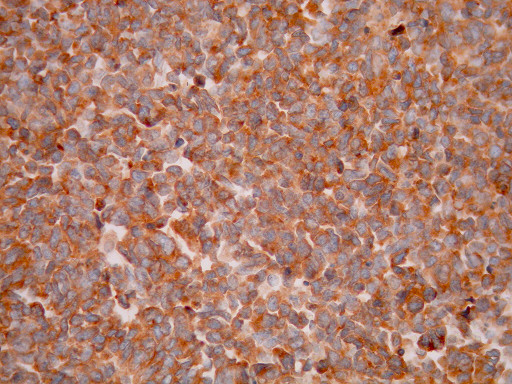
**Tumor cells are positive for Synaptophysin immunostain (×400)**.

**Figure 7:**
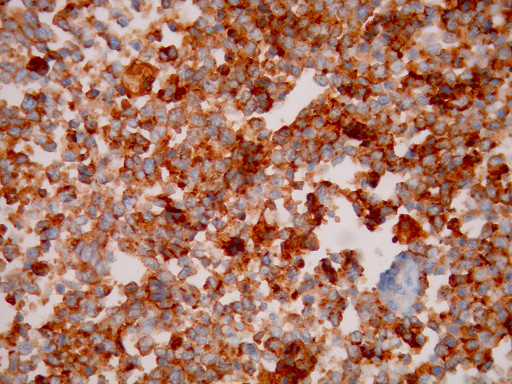
**Tumor cells are positive for CD56 immunostain (×400)**.

**Figure 8:**
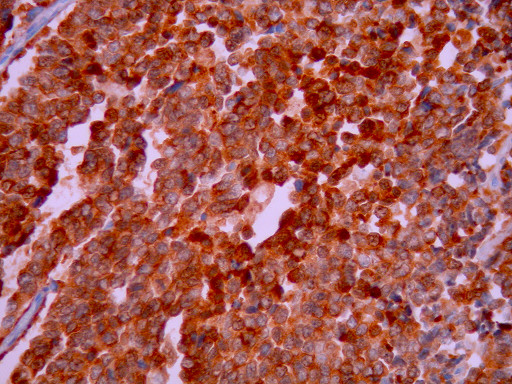
**Tumor cells are positive for NSE (×400)**.

**Figure 9:**
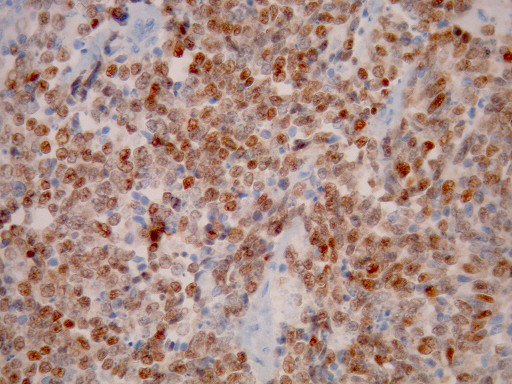
**Nuclear positivity for TTF1 immunostain (×400)**.

Histological examination showed small oval and round undifferentiated cells with oval hyperchromatic nuclei and scanty cytoplasm, as well as findings suggestive of ulcerative colitis (Figures 3, 4 and 5). Immunohistochemically the tumor was positive to synaptophysin, neuron specific enolase (NSE), CD56 and TTF1 (Figures 6, 7, 8 and 9). Based on these findings, the diagnosis was undifferentiated small cell carcinoma. The postoperative period was uneventful and the patient was discharged on the twelfth postoperative day. He received adjuvant chemotherapy with Carboplatin 400 mg/m^2 ^and Etoposide (Vepesid) 250 mg/m^2 ^and radiotherapy. Eighteen months post-surgery there is no sign of recurrence.

## Discussion

The risk of colorectal cancer for any patient with ulcerative colitis is known to be elevated, and is estimated to be 2% after 10 years, 8% after 20 years and 18% after 30 years of disease. Malignancy risk factors include extent and duration of ulcerative colitis, primary sclerosing cholangitis, a family history of sporadic colorectal cancer, severity of histologic bowel inflammation, and in some studies, young age at onset of colitis. The exact mechanism for carcinogenesis is partly unknown; in some cases it can be explained by the more widespread use of maintenance therapy and surveillance colonoscopy[3]. In our case the patient had had a history of ulcerative colitis for the last 10 years, specifically from the age of 24 years old, a condition characterized by a severe bowel inflammation.

The most common histological type of carcinoma associated with ulcerative colitis is adenocarcinoma[2]. We report this case because of the fact that SCC, instead of adenocarcinoma, on a background of ulcerative colitis is a very rare neoplasm[4]. The same factors that lead to adenocarcinoma in the background of ulcerative colitis, are also responsible for SCC, as there is the strong evidence that the tumorigenesis of all histologic types of colorectal cancer arises from a pluripotential stem cell in the mucosa of the large intestine[5]. Thus there are also other histological types of tumors arising in the background of ulcerative colitis e.g. lymphoma, lymphosarcoma, carcinoid, as the long-standing history of inflammatory bowel diseases has been proved to be responsible for carcinogenesis[6]. Still, as far as we could elicit from the literature our case is just the fifth report of SCC in ulcerative colitis[7-10], while the rarity of the histologic type of SCC in ulcerative colitis be of great interest.

Colorectal SCC is characterized by three histological types: the undifferentiated small cell carcinoma, the neuroendocrine carcinoma and the stem cell carcinoma. Each of these subtypes has different histological characteristics which reveal the differentiation of the tumor. The most undifferentiated subtype consists of small tumor cells and scanty cytoplasm. The neuroendocrine carcinoma is characterized by larger tumor cells and abundant cytoplasm. The third type is a transitional histological type between the other two types[4]. In our case, the small cell carcinoma was of the undifferentiated type.

The diagnosis of a small cell carcinoma can not be based only on the microscopical appearance, as it is often difficult to make a distinction among other "small blue cell tumors" e.g. lymphomas, melanomas, cloacogenic carcinomas[9]. For that reason the diagnosis must be confirmed immunohistochemically, as almost all types of SCC react positive to synaptophysin, chromogranin, cytokeratine and neuron specific enolase (NSE)[4]. A positive reaction to synaptophysin is the most reliable marker. However, a tumor must be positive to at least two of the markers to have a standard diagnosis[4]. In our case the tumor was positive to synaptophysin and NSE, which confirmed the diagnosis. There is a positive correlation between high differentiation of the tumor and the positivity of the above markers[4]. Nevertheless, even undifferentiated small cell carcinomas react positive to these markers, as in our case.

Primary SCC can be found in different locations such as the lung, the skin, the kidney, the thymus, the pancreas, the uterus etc[11]. The most common gastrointestinal location is the rectum, followed by the cecum and the sigmoid whereas the descending colon has never been reported as a location[4]. In our case tumor was also found in the rectum.

Small cell carcinomas are very aggressive, specifically when compared with adenocarcinomas of the same stage[4]. The 6-month survival rate is 58% and a 5-year survival rate is 6%[10]. Seventy to eighty percent of patients have already liver metastases and lymph node involvement at presentation and the prognosis is very poor[12]. For these cases multidrug chemotherapy and radiation therapy are strongly suggested[13]. Nevertheless, radical surgery offers a more favorable prognosis to some patients at an early stage, when no distant metastases are present[13]. In our case the patient had no metastases at the time of presentation, so a radical surgery was performed. Intraoperative exploration revealed no metastasis and the patient underwent a total proctocolectomy with preservation of the anal sphincters, construction of an ileal reservoir, anastomosis of the reservoir to the anus (J configuration) and protective loop ileostomy. He received adjuvant chemotherapy and radiotherapy and has no recurrence 18 months post-surgery.

## Conclusions

This is the first case of small cell carcinoma in a background of UC reported to be treated surgically and the patient and has no reccurence 18 months postoperatively.

## List of abbreviations

(SCC): Primary small cell carcinoma; (TTF-1): Thyroid transcription factor-1; (MRI): Magnetic resonance imaging; (CT)computerized tomography; (NSE): neuron specific enolase.

## Consent

Written informed consent was obtained from the patient for publication of this case report and accompanying images. A copy of the written consent is available for review by the Editor-in-Chief of this journal.

## Competing interests

The authors declare that they have no competing interests.

## Authors' contributions

All authors contributed the same.
